# Reciprocal inhibition between *miR-26a* and NF-κB regulates obesity-related chronic inflammation in chondrocytes

**DOI:** 10.1042/BSR20150071

**Published:** 2015-06-11

**Authors:** Qingyun Xie, Meng Wei, Xia Kang, Da Liu, Yi Quan, Xianming Pan, Xiling Liu, Dongfa Liao, Jinbiao Liu, Bo Zhang

**Affiliations:** * Department of Orthopaedics, Chengdu Military General Hospital. No. 270, Rongdu Avenue, Jinniu District, Chengdu, Sichuan 610083, PR China; † Department of Nephrology and Rheumatology, Chengdu Military General Hospital. No. 270, Rongdu Avenue, Jinniu District, Chengdu, Sichuan 610083, PR China

**Keywords:** free fatty acid, *miR-26a*, NF-κB, osteoarthritis, proinflammatory cytokines

## Abstract

Obesity is causally linked to osteoarthritis (OA), with the mechanism being not fully elucidated. miRNAs (miRs) are pivotal regulators of various diseases in multiple tissues, including inflammation in the chondrocytes. In the present study, we for the first time identified the expression of *miR-26a* in mouse chondrocytes. Decreased level of *miR-26a* was correlated to increased chronic inflammation in the chondrocytes and circulation in obese mouse model. Mechanistically, we demonstrated that *miR-26a* attenuated saturated free fatty acid-induced activation of NF-κB (p65) and production of proinflammatory cytokines in chondrocytes. Meanwhile, NF-κB (p65) also suppressed *miR-26a* production by directly binding to a predicted NF-κB binding element in the promoter region of *miR-26a*. Finally, we observed a negative correlation between NF-κB and *miR-26a* in human patients with osteoarthritis. Thus, we identified a reciprocal inhibition between *miR-26a* and NF-κB downstream of non-esterified fatty acid (NEFA) signalling in obesity-related chondrocytes. Our findings provide a potential mechanism linking obesity to cartilage inflammation.

## Abstract (precis)

*miR-26a* suppresses proinflammatory cytokine production via inactivating NF-κB, whereas NF-κB inhibits *miR-26a* production through binding to *miR-26a* promoter. We identified a reciprocal inhibition between *miR-26a* and NF-κB in obesity-related chondrocytes, providing a potential mechanism linking obesity to osteoarthritis.

## INTRODUCTION

Osteoarthritis (OA), a most common rheumatic disease, is characterized by irreversible destruction of the joint cartilage [1]. Obesity is causally linked to the onset and progression of this disorder [1]. Mechanistically, some studies focused on the mechanical role of obesity on OA, and other studies demonstrated that obesity-related proinflammatory cytokines or hormones largely contributed to the chronic inflammation in chondrocytes [1–3].

It is well established that obesity is accompanied with chronic inflammation, characterized by elevated proinflammatory cytokines like IL-6, TNF-α and IL-1β, as well as decreased IL-10 in circulation [4], and over-activation of inflammatory signal like NF-κB and JNK pathways in multiple cells like macrophages and adipocytes [5–7]. Obesity-induced elevation of saturated non-esterified fatty acid (NEFA) was identified as a potent inducer of those proinflammatory events [5,8]. Most recently, saturated NEFAs were also suggested to be potential proinflammatory mediators in OA [9,10], with the mechanisms being still obscure.

Proinflammatory cytokines, including IL-6 [11], TNF-α [12] and IL-1β [13], were potent inducers of OA. Especially, the IL-1 receptor has been selected as a functional target for multiple inflammatory diseases like rheumatic arthritis, in mouse models and human subjects [14,15]. Generally, the production of proinflammtory cytokines was under the tight control of several signalling pathway, among which NF-κB functioned as the central mediator [16,17]. The canonical NF-κB pathway involved heterodimers of p50/p65 [18]. Inhibition of this pathway was widely believed to have great potential as a therapeutic target in rheumatic arthritis [19].

miRNAs (miRs) are a class of ~18- to 23-nucleotied noncoding RNAs that negatively regulate mRNA stability and translation. Over the last 10 years, miRs were also identified as the regulators of inflammatory signals and played pivotal roles in the regulation of bone biology [20–24]. *miR-26a*, located in the noncoding region of *ctdspl* gene, was a recently identified microRNA, regulating inflammation response and cancer biology through directly targeting the mRNAs of a series of genes [25–28]. However, the regulators upstream of *miR-26a* were poorly characterized. Recently, a report indicated that NF-κB might regulate *miR-26a* expression to regulate cardiac fibrosis, although the precise mechanism of NF-κB-suppressed *miR-26a* expression was not fully elucidated [29].

In the present study, we for the first time identified the expression of *miR-26a* in mouse chondrocytes and deciphered a reciprocal regulation between *miR-26a* and NF-κB, providing a potential mechanism linking obesity to the production of proinflammatory cytokines in chondrocytes.

## MATERIALS AND METHODS

### Mice and diet

All the mouse experiments were conducted in accordance with the guidelines for the care and use of laboratory animals and were approved by the Animal Care and Use Committee in Chendu Military General Hospital. The male C57BL/6 mice were housed in a pathogen-free facility with a 12-h light, 12-h dark cycle and were fed with a standard chow diet (CD) or high fat diet (HFD) (D12492, fat content 60% by calorie, Research Diets, Inc.). The HFD treatment started at the age of 4 weeks.

### Isolation and culture of primary mouse and human chondrocytes

All the experiments involving human subjects were approved by the ethics committee in Chendu Military General Hospital and the informed consent was obtained from all the subjects. Human articular cartilage samples were collected from the knee joints of patients undergoing the total knee replacement surgery. Primary mouse and human articular chondrocytes were isolated and cultured following the protocol as described in our previous study [3].

### Protein extraction and immunoblotting assay

Proteins were extracted with RIPA Lysis Buffer and quantified by the BCA kit (Roche). The proteins were separated by 10% SDS/PAGE and transferred to a polyvinylidene difluoride membrane for immunoblotting assay with the antibodies [anti-GAPDH (Cell Signaling), anti-p65, anti-p-p65 (Cell Signaling)].

### Real-time PCR

Total RNAs were isolated by Trizol reagent (Invitrogen) according to the manufacture's protocol. RNAs were transcribed into cDNAs using Omniscript (Qiagen). Quantitative real-time PCR was performed using the 7900HT Fast Real-Time PCR system (Applied Biosystems). The mRNA expression levels were normalized to β-actin. Reactions were done in duplicated using Applied Biosystems Taqman Gene Expression Assays and Universal PCR Master Mix. The relative expression was calculated by the 2(^−DDCt^) method. All the primers used for PCR are available upon request.

### ChIP assay

ChIP assay was performed to investigate the interaction between the mouse p65 protein and *miR-26a* promoter DNA in the mouse chondrocytes. The detailed protocol was described previously [30]. Briefly, the primers for p65 protein-binding site were designed as: forward: 5′-GCTTAGAAAGGTAGAGGGTG-3′, reverse: 5′-ACTGACTCTCCTGCTCTGCT-3′. Amplification conditions included denaturation at 94°C for 30 s, annealing at 60°C for 30 s, and extension at 72°C for 30 s for 38 cycles, and the desired product was 105 bp.

### Gain or loss of function studies

p65 overexpression was performed by transfecting the chondrocytes with a constitutive expression plasmid for mouse p65 (empty vector pCDNA3.1 as control). Mouse endogenous p65 was knocked down by using a commercial siRNA kit. Overexpression of *miR-26a* was performed by transfecting the chondrocytes with a *miR-26a* mimic (MC10249, Life Tech.). Inhibition of *miR-26a* was conducted by transfecting the chondrocytes with a *miR-26a* mutant (MH10249, Life Tech.). For transfection of the chondrocytes, the working concentration of expression plasmids was 0.4 μg/ml, and the working concentration of siRNAs or microRNAs was 20 nmol/ml.

### Molecular cloning experiments

Those experiments for plasmid construction, cell transfection, reporter gene assays and site-directed reporter gene mutation were performed as described previously [30].

### Preparation of saturated non-esterified fatty acid solution

Stearic acid (Sigma) was firstly dissolved in the 5% fatty acid free BSA (Sigma) to obtain a stock solution (200 mM). The working concentration of NEFA was 200 μM in the present study, and the vehicle control was 5% BSA.

### Enzyme-linked immunosorbent assay (ELISA)

Cytokines in cell supernatants or plasma were measured with TNF-α, IL-1β, IL-6 and IL-10 ELISA Kits from R&D system according to manufacture's protocols. The final cytokine concentration in supernatants of cultured cells was normalized to the amount of total DNA of the cultured cells.

### Statistics

All data were expressed as mean ± S.E.M. and were analysed using either one-way ANOVA or two-tailed unpaired Student's *t*test. The difference between the groups was considered statistically significant for *P*< 0.05. For each parameter of all data presented, *P< 0.05, **P< 0.01 and ***P< 0.005.

## RESULTS

### Decreased *miR-26a* level and increased chronic inflammation in obesity associated chondrocytes

To observe the role of *miR-26a* in obesity associated chondrocytes, we set up an obese mouse model by feeding the male C57BL/6 mice with a standard HFD for 12 weeks. The HFD group compared with CD group, the body weight (Figure 1A) and plasma proinflammatory cytokines including IL-6, TNFα and IL-1β increased notably, whereas the anti-inflammatory cytokine IL-10 decreased (Figure 1B). Those results indicated that obesity was accompanied with systemic chronic inflammation. Furthermore, we verified the expression of *miR-26a* in primary mouse chondrocytes. The HFD suppressed the expression of *miR-26a* (Figure 1C) and stimulated the chronic inflammation in chondrocytes, characterized by elevated levels of proinflammatory cytokines (IL-6, TNFα and IL-1β) and decreased expression of IL-10 (Figures 1D–1G). However, the regulatory mechanism between *miR-26a* and those cytokines in chondrocytes is still obscure.

**Figure 1:**
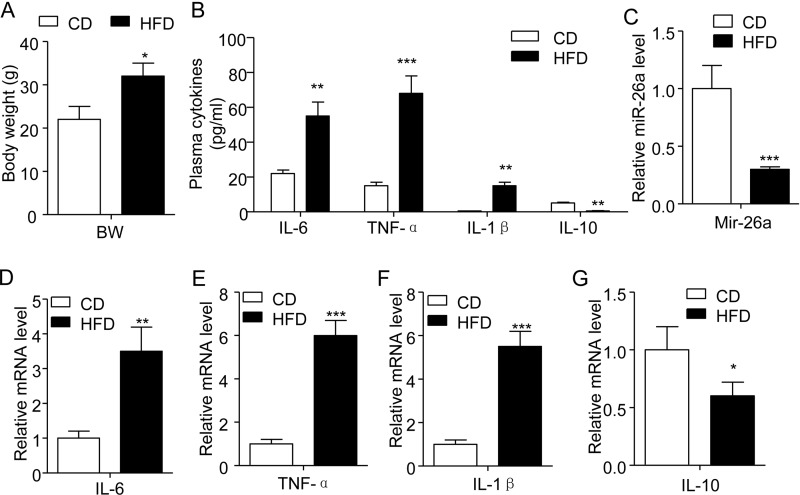
Decreased *miR-26a* level and increased chronic inflammation in obesity associated chondrocytes (**A**) Body weight (BW) of the male C57BL/6 mice fed with a standard CD or HFD (starting at the age of 4 weeks) for 12 weeks. (**B**) Plasma cytokine (IL-6, TNF-α, IL-1β and IL-10) levels of the male C57BL/6 mice fed with a CD or HFD for 12 weeks. (**C–G**) Relative mRNA levels of *miR-26a* and the cytokines (IL-6, TNF-α, IL-1β and IL-10) in the primary chondrocytes isolated from the above described mice. Data in (**A–G**), *n*=5, *P< 0.05, **P< 0.01 and ***P< 0.005.

### *miR-26a* suppresses saturated non-esterified fatty acid-induced inflammatory cytokine production in mouse chondrocytes

To explore the regulatory role of *miR-26a* in obesity-related cytokine production in chondrocytes, we employed saturated NEFA (18:0) to mimic the condition of HFD or obesity. mRNA levels of proinflammatory cytokines including IL-6 (Figure 2A), TNFα (Figure 2B) and IL-1β (Figure 2C) were notably stimulated, whereas IL-10 (Figure 2D) was inhibited by NEFA treatment in the primary chondrocytes. *miR-26a* transfection significantly diminished the aforementioned effects of saturated NEFA (Figures 2A–2D). Furthermore, the profiles of supernatant cytokines were measured and identical results were obtained (Figure 2E).

**Figure 2:**
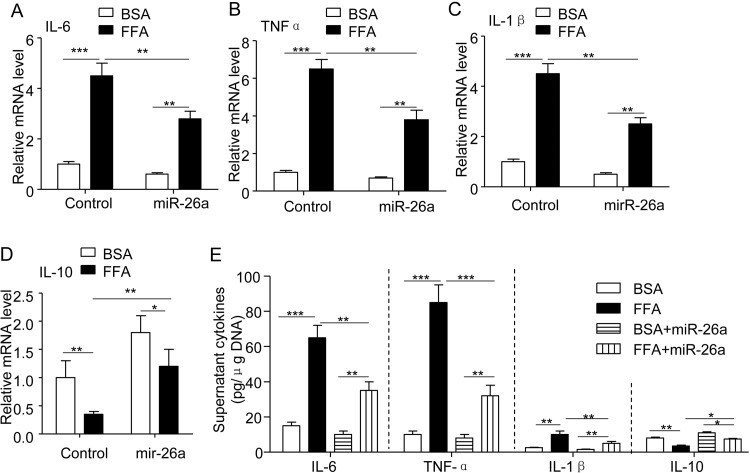
*miR-26a* suppresses NEFA-induced inflammatory cytokine production in mouse chondrocytes (**A–D**) Primary chondrocytes were isolated from 12-week-old male C57BL/6 mice and cultured *in vitro*. The cells were transfected with *miR-26a* or scramble miRNA as control for 24 h, and then treated with NEFA (200 μM) or BSA (5%) as control for 30 min. Then, the cells were harvested for the mRNA assay of IL-6 (**A**), TNF-α (**B**), IL-1β (**C**) and IL-10 (**D**). (**E**) The levels of supernatant cytokines (IL-6, TNF-α, IL-1β and IL-10) in the cultured primary chondrocytes treated as described above. Data in **A–E**, *n*=4, *P< 0.05, **P< 0.01 and ***P< 0.005.

### *miR-26a* suppresses saturated NEFA-induced NF-κB activity in mouse chondrocytes

To explore how *miR-26a* regulates proinflammatory cytokine production in chondrocytes, several inflammatory signalling pathways were scanned (data not shown). Results showed that saturated NEFA-induced phosphorylation of p65 were largely attenuated by *miR-26a* transfection (Figure 3A). In consistence, the stimulating role of NEFA in p65 phosphorylation was notably potentiated in response to *miR-26a* inhibition (Figure 3B). However, *miR-26a* did not affect the expression of total p65 (Figures 3A and 3B). To further observe the role of *miR-26a* in NF-κB activity, *miR-26a* and a reporter gene of *ccl20* promoter containing a standard NF-κB binding element were cotransfected into the primary mouse chondrocytes. Results demonstrated that saturated NEFA-induced NF-κB activity was attenuated by *miR-26a* overexpression (Figure 3C) or potentiated by *miR-26a* inhibition (Figure 3D), respectively.

**Figure 3:**
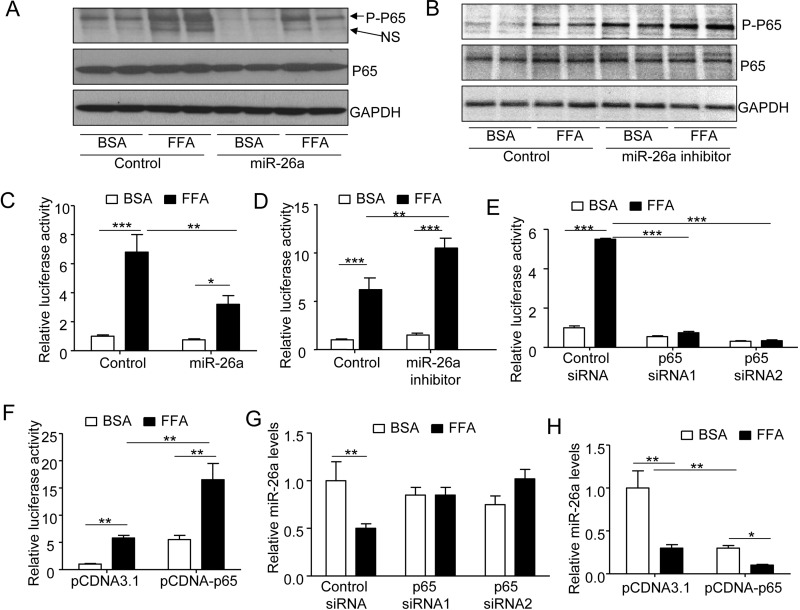
NEFA induced a reciprocal inhibition between *miR-26a* and NF-κB in mouse chondrocytes (**A**) The primary mouse chondrocytes were transfected with *miR-26a* (20 nmol/ml) or scramble miRNA (20 nmol/ml) as control for 24 h, and then treated with NEFA (200 μM) or BSA (5%) as control for 30 min. Then, the cells were harvested for immunoblotting assay of p65, phosphorylated p65 (p-p65) and GAPDH as internal control. Representative results were displayed. (**B**) The primary mouse chondrocytes were transfected with *miR-26a* inhibitor (20 nmol/ml) or scramble miRNA (20 nmol/ml) as control for 24 h, and then treated with NEFA (200 μM) or BSA (5%) as control for 30 min. Then, the cells were collected for immunoblotting assay of p65, p-p65 and GAPDH. (**C**) The primary mouse chondrocytes were cotransfected with *miR-26a* (20 nmol/ml) and a CCL20 reporter construct (0.4 μg/ml) containing a NF-κB-binding site for 24 h, and then treated with NEFA (200 μM) for 24h. Relative luciferase activity was measured. (**D**) The primary mouse chondrocytes were cotransfected with *miR-26a* inhibitor (20 nmol/ml) and a CCL20 reporter construct (0.4 μg/ml) containing a NF-κB-binding site for 24 h, and then treated with NEFA (200 μM) for 24 h. Reporter gene assay was performed. (**E**) The primary mouse chondrocytes were cotransfected with mouse p65 specific siRNAs (siRNA1 and siRNA2) (20 nmol/ml) and a CCL20 reporter construct (0.4 μg/ml) containing a NF-κB-binding site for 24 h, and then treated with NEFA (200 μM) for 24 h. The cells were harvested for reporter gene assay. (**F**) The primary mouse chondrocytes were cotransfected with a mouse p65 overexpression plasmid (0.4 μg/ml) and a CCL20 reporter construct (0.4 μg/ml) containing a NF-κB-binding site for 24 h, and then treated with NEFA (200 μM) for 24 h. The cells were harvested for reporter gene assay. (**G**) The primary mouse chondrocytes were transfected with mouse p65 specific siRNAs (siRNA1 and siRNA2) (20 nmol/ml) for 24 h, and then treated with NEFA (200 μM) for 24 h. The cells were harvested for *miR-26a* assay. (**H**) The primary mouse chondrocytes were transfected with a mouse p65 overexpression plasmid (0.4 μg/ml), and then treated with NEFA (200 μM) for 24 h. The cells were harvested for *miR-26a* assay. Data in **C–H**, *n*=4, *P< 0.05, **P< 0.01 and ***P< 0.005.

### NEFA-induced NF-κB activity suppresses *miR-26a* levels

To explore the mechanism contributing to the decreased *miR-26a* level in obesity-related chondrocytes, we observed the role of saturated NEFA and p65 on *miR-26a* production. As expected, saturated NEFA dramatically induced NF-κB activity, and this effect was blocked by p65 silence with siRNAs (Figure 3E) or potentiated by p65 overexpression (Figure 3F). Interestingly, saturated NEFA treatment notably suppressed the *miR-26a* production, whereas additional p65 silence rescued (Figure 3G) or p65 overexpression potentiated (Figure 3H) this effect. Those experiments indicated that NEFA suppressed *miR-26a* production via activation of NF-κB in chondrocytes.

### NF-κB inhibits *miR-26a* production via directly binding to an element in the promoter region of *miR-26a*

To explore how NF-κB regulates *miR-26a* production, we constructed a series of reporter genes harboring different *miR-26a* promoter sequences (Figure 4A). Reporter gene assay indicated that p65 suppressed *miR-26a* promoter activity via −2000/−1000 region (Figure 4B). By using an online software (http://www.cbrc.jp/research/db/TFSEARCH.html), we predicted several potential transcription factor-binding sites, among which the binding element of NF-κB got a high score (data not shown). By performing site-directed mutation (Figure 4C), we figured out a potential NF-κb-binding site (ggagagtccca) locating at −1947/−1937 was responsible for p65-inhibited activity of *miR-26a* promoter (Figure 4D). Further, ChIP assay verified that p65 protein could directly bind to the aforementioned binding site, and saturated NEFA or overexpression of p65 could potentiate this binding activity (Figure 4E). Thus, we concluded that NEFA inhibited *miR-26a* production through inducing the binding activity between p65 protein and the promoter sequences of *miR-26a*.

**Figure 4:**
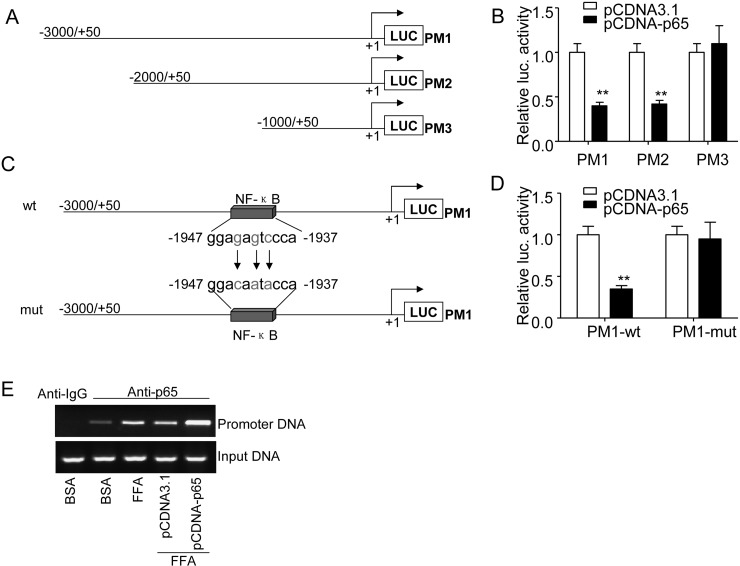
NF-κB inhibits *miR-26a* production via directly binding to an element in the promoter region of *miR-26a* (**A**) A schematic depiction of different mouse *miR-26a* promoter regions cloned into pGL4-empty vector. The constructs were designed as PM1-3 as indicated. (**B**) Effects of p65 on the promoter activity of different *miR-26a* promoter constructs. The chondrocytes were cotransfected with each promoter construct (0.4 μg/ml) and p65 overexpression plasmid (pCDNA-p65) (0.4 μg/ml) for 24 h. Then, the cells were harvested for luciferase activity assay (*n*=4, **P< 0.01). (**C**) A schematic depiction of a wild type (wt) PM1 and a mutant (mut) PM1 with a mutant NF-κB binding element located at −1947/−1937. (**D**) The wt or mut PM1 construct (0.4 μg/ml) and pCDNA3.1 or pCDNA-p65 plasmid (0.4 μg/ml) were cotransfected into the chondrocytes. Then, the cells were collected for luciferase activity assay (*n*=4, **P< 0.01). (**E**) Chondrocytes were transfected with pCDNA3.1 or pCDNA-p65 plasmid (0.4 μg/ml), and then treated with BSA (5%) or NEFA (20 μM) for 24 h. ChIP assay was performed to detect the binding activity between p65 protein and *miR-26a* promoter DNA.

### NF-κB is positively and *miR-26a* is negatively correlated with the body mass index in the patients with OA

To further correlate our *in vitro* findings to the physiopathological condition, we determined plasma NEFA levels, cartilage p65 activity, chondrocyte *miR-26a* and TNF-α levels as well as the body mass index (BMI) of the patients with osteoarthritis. We demonstrated that the plasma NEFA levels (Figure 5A), cartilage p65 activity (Figure 5B) and TNF-α levels (Figure 5D) were positively correlated with the BMI in the patients with OA, whereas the expression of *miR-26a* did the opposite (Figure 5C).

**Figure 5:**
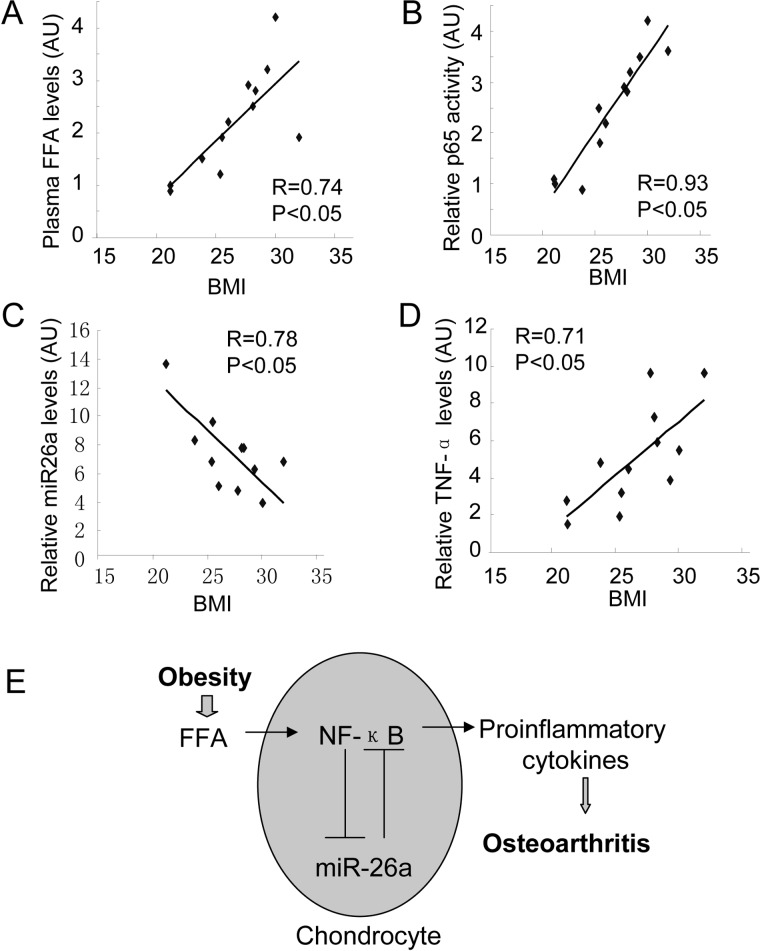
Correlation of plasma NEFA levels, cartilage p65 activity, chondrocyte *miR-26a* and TNF-α levels with the BMI in the patients with osteoarthritis (**A**) Correlation of plasma NEFA levels with the BMI in the patients with osteoarthritis (*n*=12). Pearson's correlations: *R*=0.74, and *P*< 0.05. (**B**) Correlation of p65 activity (calculated by the ratio of p-p65/p65 in immunoblotting assay) in the cartilages with the BMI in the patients with osteoarthritis (*n*=12). Pearson's correlations: *R*=0.93, and *P*< 0.05. (**C**) Correlation of *miR-26a* levels in the chondrocytes with the BMI in the patients with osteoarthritis (*n*=12). Pearson's correlations: *R*=0.78, and *P*< 0.05. (**D**) Correlation of TNF-α mRNA levels in the chondrocytes with the BMI in the patients with osteoarthritis (*n*=12). Pearson's correlations: *R*=0.71, and *P*< 0.05. (**E**) Proposed models for reciprocal inhibition between *miR-26a* and NF-κB regulating obesity-associated chronic inflammation in chondrocytes. Briefly, *miR-26a* exerts inhibitory effect on NF-κB activity and proinflammatory cytokine production in chondrocytes. In obesity, elevated NEFA in circulation might stimulate NF-κB activity, which suppresses *miR-26a* production and further potentiates the production of proinflammatory cytokines. Those elevated proinflammatory cytokines from the chondrocytes aggravate the inflammation in cartilage, perhaps inducing osteoarthritis.

## DISCUSSION

In the present study, we are the first to report the expression of *miR-26a* in mouse chondrocytes and to identify NF-κB (p65) as a direct suppressor of *miR-26a*. Interestingly, *miR-26a* could also suppress proinflammatory cytokine production via inactivating NF-κB. Thus, we provided a potential mechanism linking obesity to the onset and progression of OA (Figure 5E).

Obesity is causally linked to OA, with the mechanism not fully understood. Saturated NEFAs were well established as an initiator of chronic inflammation and insulin resistance in obesity [5,8]. General studies focused on the role of NEFAs in macrophages or insulin-responsive tissues like adipose tissue, liver and muscle [5,8]. In the present study, we identified saturated NEFA (18:00) was also a potent inducer of chronic inflammation in chondrocytes. In detail, NEFA could stimulate proinflammatory cytokine production via induction of NF-κB activity and suppression of *miR-26a* production in chondrocytes. These findings explained well how obesity caused chronic inflammation in chondrocytes. It should be pointed out that transcription factor NF-κB commonly functioned as an inducer of target genes [31], whereas in the present study we identified it as a suppressor of *miR-26a* by directly binding to the promoter region. However, the detailed mechanism need further to be explored.

miRNAs are small (~22 nucleotides) non-coding RNAs that bind to complementary sequences in the untranslated regions of target mRNAs and contribute to gene regulation by reducing mRNA translation or destabilizing transcripts [32–34]. The commonly accepted mechanism of miRNA regulation is that the seed region (2–8 nucleotides at the 5′ end) of a miRNA is complementary to the 5′ or 3′ untranslated region (5′- or 3′-UTR) of an mRNA, leading to mRNA degradation or translational inhibition [32,33]. Recent work has shown that miRNAs have multiple effects in various tissues including chondrocyte inflammation [22–24]. Generally, production of proinflammatory cytokines is tightly controlled by several signalling pathways, among which NF-κB was the most important one [16,17]. In the present study, we found that *miR-26a* could inhibit NF-κB activity by suppressing the phosphorylation of p65 in mouse chondrocytes. Unfortunately, we could not verify the direct interaction between *miR-26a* and p65 gene. Thus, we presumed that *miR-26a* regulated p65 activity in an indirect manner.

Significantly, we demonstrated that the plasma NEFA levels, cartilage p65 activity and TNF-α levels were positively, whereas the expression of *miR-26a* was negatively correlated with the BMI in the patients with OA. Those findings verified the reciprocal inhibitory role between NF-κB and *miR-26a*. Meanwhile, the BMI would be a promising indicator of chronic inflammation in the chondrocytes or cartilage. Hypolipidemic treatment might be an efficient strategy to decrease the obesity-related proinflammatory cytokine production in chondrocytes.

In conclusion, reciprocal inhibition between *miR-26a* and NF-κB downstream of saturated NEFA signal regulates obesity-related chronic inflammation in chondrocytes. Our findings provide a potential mechanism linking obesity to cartilage inflammation.

## Abbreviations

BMI: body mass index
CD: chow diet
NEFA: non-esterified fatty acid
HFD: high fat diet
miR: miRNA
OA: osteoarthritis
